# A qualitative study of experiencing cauda equina syndrome and its aftercare in the UK

**DOI:** 10.1038/s41393-025-01097-7

**Published:** 2025-06-28

**Authors:** Nisaharan Srikandarajah, Simon Clark, Martin Wilby, Tony Marson, Adam Noble

**Affiliations:** 1 Institute of Systems, Molecular & Integrative Biology, Faculty of Health and Life Sciences, University of Liverpool, Liverpool, UK; 2 Department of Neurosurgery, The Walton Centre NHS Foundation Trust, Liverpool, UK; 3 Institute of Population Health Sciences, Faculty of Health and Life Sciences, University of Liverpool, Liverpool, UK

**Keywords:** Quality of life, Outcomes research

## Abstract

**Study design:**

Qualitative, semi structured interviews.

**Objectives:**

Cauda Equina Syndrome (CES) is a neurological emergency that can cause permanent disability to the lower limbs, including pain, weakness, and bladder, bowel and sexual dysfunction. There is little evidence on the lived experience of patients with different severities of CES. This study sought to address this.

**Setting:**

The interviews were conducted with persons who had experienced CES and been operated on for this condition in the UK.

**Methods:**

A sampling frame was used on a pre-existing database to select a maximum variation sample. Interviews were audio recorded and transcribed for thematic analysis supported by NVivo.

**Results:**

Data saturation was achieved with 22 patients (12 female, 10 male) of whom 10 had CES-incomplete and 12 had CES-complete. Average age was 46 years and time since the operation was 62 months. Most interviews took place at the patients’ home or workplace. Data analysis identified 4 main data themes: (1) Varying priorities of physical health; (2) A fragmented healthcare service; (3) The process of adjustment; and 4) Anticipatory anxiety and diminished sense of self-worth.

**Conclusion:**

The identified themes confirm that CES can be a chronic condition, which requires holistic support to address the long-term outcomes. This highlights the importance of using the Cauda Equina Syndrome Core Outcome Set (CESCOS) in CES research studies to record these outcomes.

## Introduction

Cauda equina syndrome (CES) is a serious neurological condition, which in most cases requires emergency surgical intervention. It occurs when the cauda equina nerves are compressed, be it due to a herniated intervertebral disc, spinal stenosis or trauma. Its most severe presentation, referred to as CES complete (CESR), involves the person presenting with painless urinary retention with overflow incontinence and complete perianal sensory loss. CES incomplete (CESI), on the other hand, involves the person presenting with symptoms such as urinary issues of neurogenic origin including loss of desire to void, altered urinary sensation, and hesitancy with partial saddle anaesthesia. In England, CES has an incidence of ~2:100,000 [1, 2] and can affect people at the peak of their working lives.

Little evidence is available on what it is like living with CES after acute treatment. Such information is crucial for ensuring clinical services are designed to meet the needs of patients.

Qualitative methods allow patients to raise what *they* regard as important aspects and concerns rather than these being specified in advance by the researcher.

One qualitative study [3] interviewed 11 patients with CES, and provided insights into what it is like living with CES. Three themes emerged – namely, “dissatisfaction with care”, a “struggle to gain social identity in relation to having a ‘hidden’ disability” and “renegotiating identity following CES”. This study focused on people with CESI due to a prolapsed lumbar disc over the short term (2 or fewer years). Thus, what the longer-term picture is like remains unclear.

To address these limitations and provide a more comprehensive understanding, we conducted a qualitative study of the experiences of people with CES from a variety of causes, both CESI and CESR, in the medium and long-term.

## Methods

### Design

Semi structured qualitative interviews were conducted with adults who had experienced CES. The research paradigm adopted was pragmatism [4].

Persons were interviewed as a part of a wider international consensus project to identify the minimum core set of outcome domains, to be used by all trials evaluating interventions for people with CES [5]. The primary purpose of the interviews was to identify outcome domains of importance to CES patients [6]. However, since the language of ‘outcomes’ can be unfamiliar, patients were invited to provide a chronological narrative of their experience with CES, undergoing treatment and life afterwards. This was reanalysed for the purpose of the present study.

### Procedure

Following Riessman [7], an interview topic guide was developed based on the literature and refined through the iterative process of conducting 2 pilot interviews (Table 1).

**Table 1:** Themes and related questions in topic guide.

Themes	Example question
Severity of CES	What were your expectations of life health-wise after the operation? What was the reality like?
Duration since operation	Due to this condition what do you feel are the challenges to your health and wellbeing? Have these changed with time?
Hospital care	How do you feel your condition has been managed in hospital and in the community?
Aftercare	Tell me a about the support you had for the condition? Is there anything you would have liked to change?

NS (MBBS FRCS PhD), a male Specialist Trainee in Neurosurgery with training in qualitative methods, conducted the interviews. Participants were kept anonymous and told the study results would be published. Interviewees were informed at the introduction that the interviewer was part of the research team.

To promote transparency interviews were audio-recorded and subsequently transcribed verbatim and checked for accuracy by NS. Transcripts were not returned to participants for comment or correction.

### Recruitment and setting

Persons were eligible to participate if they: had received a diagnosis of CES (any type); were aged ≥18 at the time of the study; had undergone a surgical procedure for CES less than 10 years previously; and were able to provide informed consent and participate in an interview in English.

We aimed to recruit until data saturation was achieved. Based on prior studies we anticipated this might occur after 15 to 40 interviews [8, 9].

Participants were identified and invited with the support of a tertiary neurosurgical centre in North-West England. Participants who had undergone spinal surgery to remove a compressive lesion for CES between 2005 and 2015 were recorded on an internal database by the medical team. The centre was provided with a stratified purposeful sampling framework [10] to identify persons from this database for invitation. People from the database were randomly selected and categorised according to whether it was ≤2 years since their CES (“short”) or 2 years to ≤10 years (“long”) and whether they had a CESI or CESR. This was created to reduce the selection bias of having the people with the most severe condition (CESR) for the longest duration only responding.

Each selected participant was posted an invitation from the centre. Recipients could return an opt-out response if not interested in participating. Willingness to participate, verifying eligibility, and, if applicable, arranging an interview were done. The study is reported in line with the Consolidated criteria for Reporting Qualitative research (COREQ) [11].

### Consent

All participants provided informed consent. The National Research Ethics Service Committee (REC reference 16/SC/0587) and Health Research Authority (IRAS Project ID 201946) approved the study.

### Analysis

Thematic analysis, informed by Braun and Clarke [12], was used to enable scrutiny of data across the sample and within individuals’ transcripts. It was conducted deductively with the identification of pre-existing themes underpinned by previous research and clinical experience and inductively with the identification of themes grounded in the data [13] to identify patterns.

QSR International’s NVivo V.12 qualitative data analysis software was used as a management tool throughout the process. Nodes were created to mark relevant concepts and topics in the text documents. Relationships between themes were identified through constant comparison of the transcripts, nodes, and categories. Quotations are presented to illustrate themes.

## Results

### Participants

Of the 100 patients sent invitations, 15 patients declined participation, mainly by returning an “opt out” sheet, without providing a reason.

Ultimately, data saturation was judged to have been achieved after 22 interviews. The sample was comprised of 12 females and 10 males (Table 2). Ten had CESI and 12 had CESR. Participants’ average age was 46 years (range 31–61, standard deviation [SD] 9.21). The mean number of operations the participants had was 1 (range 1–4, SD 0.8), whilst average time since having the operation was 62 months (range 4–122, SD 38.1).

**Table 2:** Demographics and clinical details of participants and interview details.

ID	Sex	Age	Time since operation	Operations (n)	Type	Mins	Location
1	M	50	7 years, 5 months	1	CESI	48	Home
2	M	49	6 years, 2 months	1	CESI	59	Work
3	F	35	8 years, 4 months	2	CESI	52	Home
4	F	35	7 years, 6 months	4	CESR	51	Home
5	F	57	2 years, 6 months	1	CESI	27	Phone
6	F	47	1 years, 2 months	1	CESI	28	Home
7	M	38	7 years, 4 months	2	CESI	50	Hospital
8	F	31	8 years, 4 months	2	CESI	48	Home
9	F	56	0 years, 9 months	1	CESR	38	Home
10	F	40	0 years, 10 months	1	CESI	33	Home
11	F	46	2 years, 6 months	1	CESR	64	Home
12	M	59	5 years, 4 months	1	CESI	44	Skype
13	M	44	7 years, 1 months	1	CESR	33	Home
14	M	47	0 years, 4 months	1	CESR	46	Home
15	F	58	10 years, 2 months	1	CESR	39	Home
16	M	56	6 years, 4 months	2	CESR	43	Hospital
17	F	46	9 years, 3 months	3	CESR	72	Home
18	M	61	9 years, 1 months	1	CESR	54	Home
19	F	42	7 years, 2 months	1	CESR	30	Home
20	F	36	7 years, 3 months	1	CESR	54	Home
21	M	32	3 years, 11 months	1	CESR	27	Home
22	M	50	1 years, 6 months	1	CESI	52	Home

*CESI* cauda equina syndrome incomplete, *CESR* cauda equina syndrome with urinary retention.

Interviews had a mean length of 45 min (range 27–72, SD 12.3). Most (*n* = 18) took place at the patient’s home or workplace. The remainder occurred by phone (1), by video conferencing (1) or in person at the centre (2). For 20 interviews, only the patient was present. For the remaining 2, the patient was, at their request, accompanied by a spouse/partner.

### Themes

The raw data from the transcripts was coded and then placed into domain summaries. Domain summaries were higher order groupings that collectively summarised what the similar outcomes were describing. Table 3 shows the domain summaries and the ideas for provisional themes, which led to the development of the 4 final themes.

**Table 3:** Domain summaries, ideas and themes.

Domain summaries	Ideas	Themes
Prioritisation, bladder, bowel, sexual, musculoskeletal, fatigue, postural difficulties, stiffness, back and leg pain, sensation	Varying priorities (of outcomes)- depending on severity	1. Varying priorities of physical health
Delay in management, follow up	Anger/ discontent over disjointed management/ feeling fragile/ sudden change in circumstances/ lack of follow up, services and holistic care	2. A fragmented healthcare service
Returning to work, support, recovery	Process of adjustment- support/awareness, reduced opportunities, work, recovery	3. The process of adjustment
Anxiety, isolation, low mood, suicide, reasoning and awareness	Anxiety over future prognosis/ outcome, physical struggle to improve, diminishing importance, reasoning for acceptance, feeling like a “fraud” “not believed.” Isolation, low mood	4. Anticipatory anxiety and diminished self-worth

This study is reported in line with COREQ (Supplementary Appendix 1) and the Springer Nature reporting reproducibility checklist was completed (Supplementary Appendix 2).

#### Theme 1: Varying priorities of physical health

Patients had varying levels of prioritisation depending on the severity of their condition. Generally, CESI patients felt bladder and bowel outcomes had the most impact whilst for CESR patients it was mobility issues and pain control.

*“My bladder and everything to do with my ‘plumbing’ would be number one… I would still be able to look after myself pretty much and take medication for the leg pain, but the thought of losing all that and being dependent on other people that would be like a nightmare”*

(Participant 22: M, 50, CESI, time since initial operation 1 y 6 m)

*“If you can get the pain under control then you can deal with everything else. Pain, mobility, bladder and bowel yes, they’re the ones that are the most important in that order … I would much rather have a colostomy bag and retain the ability to move around, walk, interact socially and work”*

(Participant 14: M, 47, CESR, time since initial operation 4 m)

Patients with bladder dysfunction reported a range of changes including urinary frequency, urinary retention, overflow incontinence, and an inability to feel when they passed urine. CESR patients who continued to experience bladder and bowel difficulties reported frustration, embarrassment, and negative effects on their intimate relationships. The main issue causing loss of physical sexual intimacy and emotional distancing between partners over time for male participants was with the inability to achieve or maintain an erection. For women, the issue was due to numbness in the saddle area.

Fatigue was commonly reported by participants and how the “effort” of doing an activity was greater than before. To recover extended periods of sleep and rest were required. This led to greater or complete assistance from family and friends with household duties.

Back pain was intense for many CES patients and described as “*exhausting*,” “*over-rode everything*,” “*suicide pain*,” “*back was like a rusty hinge*,” and like “*sticking a knife in your back*.” Significant back pain and stiffness “*like someone had opened my back and poured lead in there*” after the onset of CES could limit them standing upright, walking, and sleeping. It also impacted on home and workplace activities.

#### Theme 2: A fragmented healthcare service

Many patients in the study reported a time delay between their initial symptoms and undergoing imaging (e.g. MRI) to diagnose their CES. Many recalled making multiple trips to care providers before imaging was organised and receiving their diagnosis. They reported frustration and a perception that there is a lack of awareness amongst healthcare professionals (HCPs) regarding CES.

*“There are an awful lot of points there where it (CES) should have been picked up. If I had ended up like some people, I would have probably taken that route (legal action) because I do genuinely feel that this syndrome (CES) is not taken seriously”*

(M, 49, CESI, 6y2m, participant 2)

Surgery was reported by most patients to relieve or reduce the severe leg pain but medical follow up was described as unsatisfactory by almost all participants. They reported receiving conflicting or incorrect information regarding CES. They described anxiety over what they could and could not do physically.

*“There wasn’t a real follow up from the hospital other than the three-month questionnaire… but what I still don’t know is it going to get worse, am I doing the right thing by walking … am I pushing it to the limit, is that ok. Should I be resting?… I still don’t know if I am doing the right thing or not”*

(M, 50, CESI, 1y6m, participant 22)

#### Theme 3: The process of adjustment

Reduced mobility due to back and leg pain was the most common reason to be unable to continue employment. Some patients had returned to work with appropriate adaptations made for them, but most employers were described as not making adaptations and instead often recommending early retirement for medical reasons.

Generally, patients who returned to work with supportive staff valued the routine despite the difficulties. In all cases where patients were unemployed, they missed their jobs as they had derived significant satisfaction from their role.

*“Previously I had been very active … so not being able to work and do something that you enjoy … that’s what put me in this place of isolation and depression because it is suddenly so much activity to nothing at all”*

(F, 57, CESI, 2y6m, participant 5)

The informal caring support from family and friends was described as more consistent and reliable than that received from the formal health service. Primarily, the patient’s partners played a significant role in caring for them in the short term after the operation to longer term care like housework and exercise. There was a lack of experience of formal support groups amongst most participants.

Generally, participants described that if there was any recovery with their bladder or bowel function that it had occurred within 2 to 3 months of the operation. Back and leg pain caused by CES were the most obvious features to patients hence when this resolved after the operation it was a great relief to them.

#### Theme 4: Anticipatory anxiety and diminished self-worth

A substantial proportion of CES patients reported being worried about their prognosis, physical health and future employment. They attributed this to not being clear on the cause of their condition, what to do after the operation, including what physical activities were safe. Some felt the process of hospital admission, diagnosis, surgery, and discharge within a short space of time was “like a trauma”, impacting them, psychologically.

*“It just worries me as I get older am I going to end up in a wheelchair because I’m in that much pain … and I’m thinking job wise how long have I got left in this job?”*

(F, 42, CESR, 7y2m, participant 19)

*“I’m very grateful that I can walk, and I have the sensations back but I feel a little bit like a time bomb that another part of the disc could go at any point”*

(F, 31, CESI, 8y4m, participant 8)

Isolation was described by many. They attributed this to a variety of factors ranging from a lack of access to effective and regular health care support and that some HCPs were perceived as often being dismissive of the challenges they were undergoing. Bladder and bowel dysfunction contributed to the feelings of ‘aloneness’ due to the fear of having “accidents”. The physical difficulty of having sex in some instances made patients feel more distant from their partners, with relationships ending in some instances.

Low mood, and to a lesser extent suicidal ideation, was reported by a few participants. They said they had struggled to cope at work due to the back and leg pain and reduced mobility and some had their jobs terminated prematurely. Two participants reported that psychological distress culminated in them attempting suicide as they were dealing with the consequences of CES and significant personal events at the same time.

Generally, people with CES realise there low public awareness regarding the condition. CESI participants are aware of the range of more severe outcomes that they may have experienced and were grateful they did not.

“*The residual nerve damage is always there and the way I look at it it’s a small price to pay for what I believe other people have suffered a lot worse than what I have*.”

(M, 50, CESI, 1y6m, participant 22)

There was interest and determination amongst many patients to pursue exercise, but they had anxiety over the long-term effects. Those who are reassured by HCPs try to do core building exercises like pilates, swimming and walking.

## Discussion

### Main findings

This is the first qualitative study to explore the lived experience of CES patients according to the severity of the condition (CESI and CESR) in the medium to long-term. Participant’s experiences of living with CES and its consequences were captured by 4 main themes detailed below. The ideas and themes mentioned reflect many of the outcomes in the CES core outcome set (CESCOS) (5). These were decided as the most important outcomes in CES to be included in all future research studies by an international consensus through a Delphi process and consensus meeting involving patients and healthcare professionals.

### Varying priorities of physical health

In our interviews, people with CES were concerned with their sense of self and identity, especially with regards to autonomic dysfunction (e.g. bladder, bowel, sexual issues) or mobility. They wanted to remain as ‘normal’ as possible to the outside world, whilst at the same time wanting others to understand that they had a disability. This challenge of conflicting identities has also been identified by Hall and Jones [3]. Their study only interviewed CESI patients and found that bladder and bowel dysfunction were overall the most concerning symptoms reported by patients. We interviewed people with different severities of CES. CESI participants identified bladder and bowel function as the most important symptoms. CESR patients though, prioritised pain and mobility. What was also novel were the changes reported by patients over time. For instance, initially pain was said by most patients, regardless of severity, as being overwhelming before the operation, but that numbness, foot drop, stiffness, and mobility became a concern after the operation. Bowel and bladder issues could, in some instances, also become normalised over time, whereas achieving normalisation of pain remained difficult.

Although not overtly mentioned in the medical setting and literature [14, 15] it is evident from this study that sexual function is an issue which is crucially important to CES patients. Fatigue had also disrupted certain CES participants daily home or work routine, quality of life and social interaction, which has not been mentioned or reported previously in the CES literature.

Participants felt that they received minimal support after treatment for CES and underwent a “trial and error” period of learning how to manage their pain, which highlights how pain should be managed as a priority in CES.

### A fragmented healthcare service

In our study, whilst patients were complimentary regarding their surgical treatment and relief of acute symptoms, they were more critical regarding the diagnosis and aftercare provided.

The communication from HCPs was reported as not being clear enough regarding the “red flag” signs of CES, which led to anger and frustration at the delayed diagnosis and unsatisfactory aftercare received especially by CESR patients with more severe symptoms. Research in chronic fatigue and pain has revealed that when symptoms are not visible or harder to measure they risk being dismissed by HCPs and society leading to patient distress and anger [16]. Patients in this study felt HCPs in primary care and emergency departments did not take their concerns seriously enough. This could be ascribed to CES being clinically difficult to differentiate from the more common lower back pain or leg pain, which does not require immediate intervention. It is difficult to justify acute investigations in a healthcare system with limited resources when most back and leg pain improves with conservative management [17].

The short follow up and discharge for CES patients from spinal services explains why many of our participants described relying on close family and friends network for support. The combination of having a bad clinical outcome, feeling unsupported and being encouraged by medico legal companies to file a complaint seemed to contribute to litigation.

It is clear from this there needs to be a holistic service for CES patients, which focuses on long term care and management rather than acute management and discharge. This has been seen in other neurological conditions, for example in patients who have been treated for subarachnoid haemorrhage where post discharge services were not addressing long term needs [18]. A consideration would be to position management of CES long term issues such as neuropathic pain with appropriate extra resources and funding within the remit of SCI rehabilitation services where these services already exist.

### The process of adjustment

Although there is a lack of evidence regarding employment in CES, the impression is that when the condition is more severe (CESR) the employment opportunities decrease. Actively contributing as a member of society is valued highly by most people with disabilities [19] and there is strong evidence to suggest that it is better for an individual to work than not [20]. CES patients in our study were satisfied when they returned to work with the necessary adaptations being made with their pre-injury employer. There were indications from our data that the process of adjustment to a meaningful routine had been longer or remained unresolved for those CES patients who were unable to return to work.

In our study, there was an interest amongst patients to pursue exercise, but this was tempered by a lack of knowledge as to what was clinically safe. Clinicians should therefore encourage moderate exercise for CES patients.

The lack of guidance and goal setting is evident in CES where patients have expected recovery to normal and become very disappointed when they do not reach this. There needs to be realistic goal setting in the aftercare for CES patients depending on the severity of their condition.

### Anticipatory anxiety and reduced self-worth

There is little within the literature regarding the difficulties CES can have for a patient’s mental health [3, 21].

Feelings of low mood, suicidal ideation, isolation and anxiety have been explored in detail in this study. Patients mentioned a lack of adequate guidance, follow up, support services and appropriate pain management contributing to these feelings. The use of a psychiatrist could help resolve the mental issues patients experience. Improving understanding of CES patients and setting realistic goals, as mentioned before, could lead to improved outcomes and re-integration with the wider society.

### Potential strengths and weaknesses

Strengths of this study include reporting and conducting according to best practice (e.g., COREQ). It is also the only study to look at both CESI and CESR patients to date. The only other qualitative study available is by Greenhalgh et al. [21] and its focus was on the initial presentation of CES and did not categorise severity. Weaknesses in this study was that the sample is from the North West region in the UK only but UK has universal healthcare so management is expected to be similar in other regions.

## Conclusion

CES has always been managed as acutely in the healthcare system to reduce the risk of serious neurological injury. The lack of out of hours MRI services adds to the delay in diagnosis and should be part of the resource planning. Hospital and community services are not equipped to deal with the longer term medical and psychological consequences of this condition. Patients tend to find their own solutions with varying levels of success without access to the appropriate services, and potentially significantly delays recovery. Not only does this confirm the importance of using the core outcome set for future research in CES [5] but also highlights the need for a more holistic service for CES to appropriately manage the longer-term effects. This may involve more constructive structured interaction with physiotherapists, psychologists, relevant medical/ surgical specialties and other CES patients through support networks. This would allow the full benefits and costs of different treatment options to be clearly identified and compared in future studies.

### Reporting summary

Further information on research design is available in the Nature Research Reporting Summary linked to this article.

## Supplementary information

COREQ Checklist

Reporting Summary

## Data Availability

The data for this study is available within this published article.
